# Unintended Positive Consequences of Development Centres in University Graduates

**DOI:** 10.3389/fpsyg.2021.775377

**Published:** 2021-12-01

**Authors:** Melissa White, Jürgen Becker, Marieta du Plessis

**Affiliations:** Department of Industrial Psychology, University of the Western Cape, Cape Town, South Africa

**Keywords:** development centres, graduate employability, self-efficacy, competency-based assessments, social cognitive theory

## Abstract

This study investigated development centres as a method to improve the generalised self-efficacy of university graduates. This research was motivated by the various challenges, graduates face in order to successfully transition into the world of work. Although there is a general scarcity of skills in many emerging economies like South Africa, graduate unemployment rates remain high. Additionally, graduates are not making the immediate impact that employers would expect due to a lack of technical and “soft skills.” General self-efficacy is an important attribute for job applicants because it provides them with the confidence to solve problems efficiently. The primary research objective was to identify whether the generalised self-efficacy of graduates can be positively affected by a development centre approach in the short-term and long-term. The sample population for this research included Industrial Psychology graduates at a select university in the Western Cape, South Africa (*n*=17). A quasi-experimental methodology was implemented where an intervention group (*n*=7) and a control group (*n*=10) were taken through a development centre approach. The results of the intervention indicated that a development centre approach has a positive impact on self-efficacy levels over the short and medium term. Results from the study emphasise the importance of self-efficacy in graduate employability and indicate how development centres can be used to improve self-efficacy levels. The findings of this study provide a basis for future research into the further development of graduate self-efficacy and the potential benefits for first time job seekers.

## Introduction

One way of reducing poverty is by increasing employment rates throughout the various sectors of the economy. Voluminous research suggests that a fast growing and inclusive economy presents the best long-term strategy to roll back income inequality, unemployment, and poverty (Hoeller et al., 2014; Sulla and Zikhali, 2018). For this reason, the South African National Development Plan (NDP) aims to meet its laudable 2030 goals by providing more broad-based employment through faster economic growth, improving the quality of education, and building a capable state (National Planning Commission, 2012). However, several structural challenges hamper economic growth and broad-based employment including low investment in basic infrastructure, inflexible labour laws, low levels of labour productivity, and the high cost of doing business in South Africa. These trends have limited the ability and willingness of the private sector to employ graduates produced by the tertiary education sector over the last 20years (Tremblay et al., 2012; Humburg et al., 2013; Mlambo et al., 2021). Although South Africa has always been characterised as a country with high unemployment rates, especially amongst semi-skilled or unskilled workers, this trend is now extending to skilled employees as well. Statistics South Africa (2021) reports growing unemployment rates of 34.4% in quarter 2 of 2021, with the burden of unemployment squarely shouldered by the youth (aged 15–34years). The report goes on to reveal substantially higher unemployment rates for youth, amounting to 64.4% for youth aged 15–24, and 42.9% for those aged 25–34. It also suggests that even graduates of tertiary education programs are not guaranteed work in South Africa, with 11% of graduates finding themselves unemployed in Q2 2021 (Statistics South Africa, 2021). This presents a developmental issue not only faced by students, but also by organisations, tertiary institutions, and the nation as a whole (Okeke-Uzodike and Naude, 2018).

Developing employable graduates who display desired graduate attributes is an important starting point in reducing the high unemployment rates of graduates. As previous research has indicated, organisations seek graduates with a combination of skills, such as problem solving skills, leadership skills, interpersonal skills, and communication skills (Lowden et al., 2011; Awan and Ameen, 2020). However, with the lack of exposure to the working world, graduates may not have access to development opportunities to improve their employability. Du Preez et al. (2019) recommend the necessity for metacognitive competence development to help graduates develop an understanding of their own skills and what is required in the workplace. Hamilton et al. (2015) add by stating that only when the development of practical and fundamental skills is prioritised will graduates become employable.

Developing confidence in graduates is an important step in preparing them for the challenges and opportunities represented by the world of work. When graduates have a high level of self-efficacy, they are more courageous in taking risks, they make better decisions, and they are able to tackle challenges efficiently, and can set stimulating goals and commit to them. They also experience lower levels of stress, anxiety, and depression, and put more effort and perseverance into learning challenging tasks (Luthans and Avolio, 2007; Petruzziello et al., 2021). As a result, graduates can benefit from impactful efforts at improving self-efficacy and employability, such as coaching and mentoring programmes, work-integrated learning methods (Freudenberg et al., 2010), job shadowing, competency-based curricula (Muraraneza et al., 2017), and development centres. While the competencies that these methods of developing graduate employability are pivotal to students’ future professional success, the underlying benefit of this development – self-efficacy – is also important to their personal growth and maturity.

### The Role of Higher Education in Developing Graduate Self-Efficacy

According to Brits (2018), tertiary institutions play a critical role in both enhancing graduate employability, and in impacting the national economic growth. Education plays a pivotal role in the NDP of 2030, to spur growth, and broaden employment opportunities. In this regard, higher education in South Africa plays a key enabling function. Moolman (2017) states that tertiary institutions can assist graduates in achieving employability by embedding this concept in learning programmes and lectures, incorporating work experience into the academic curriculum, and introducing development assessment practices. One way in which tertiary institutions can contribute to this transformation is by introducing elements of development centres into their teaching practice, since development centres offer numerous benefits that may improve academic performance and employability (Van Wyk and du Toit, 2018). Jacobs et al. (2018) state that graduates benefit from assessment centres regardless of the career they aspire towards, and are provided with structured feedback that guides their steps towards future development. A longitudinal study by Jacobs et al. (2018) found that over a 10-year period, assessment centres developed the skills and abilities of all graduates who participated, irrespective of their position. Although the primary goal of development centres is to diagnose the key strengths and weaknesses of participants, one of the most important and overlooked benefits is the development of generalised self-efficacy.

Assessment and development centres can thus play a critical role in nurturing the competencies crucial to job success by creating self-awareness and boosting self-efficacy. This study, therefore, proposes that development centres have the potential to increase self-knowledge and awareness regarding the strengths and weakness of participants, while boosting self-efficacy in the short and long term. This in turn is likely to improve the employability of students and assist them to make an immediate impact in the organisations that employ them.

The next section will look at the theoretical basis of generalised self-efficacy generally and, more specifically, at the links with competency-based assessments.

## Theoretical Background and Hypotheses

### Graduate Employability

Graduate employability can be simply described as the degree to which graduates possess the knowledge, attributes, skills, and attitudes to attract interest from employers, and ultimately to provide meaningful contributions to the workplace and the broader economy (Tomlinson and Nghia, 2020). While many employability models have been developed, Understanding, Skills, Efficacy, and Metacognition (USEM) model of Knight and Yorke (2002) has gathered substantial support from contemporary researchers in the area (see Soares et al., 2017; Bennett and Ananthram, 2021). The essence of the USEM model is that it requires more than just generic skills or a qualification to be considered employable, and that these components are interdependent.

An additional employability framework developed by Dacre Pool and Sewell (2007), known as the CareerEDGE model shows the foundation required for employability, along with the direction and relationship of further competencies required from graduates to develop their employability. The foundation of this model displays a degree of competency overlap, for example, work experience may be essential to career development learning, but may also inform degree subject learning relevant to an individual’s course of study. It is thus evident that although self-efficacy plays a crucial part in graduate employability, so does the access to development opportunities as represented in the five aspects of the CareerEDGE model foundation. Implementing creative opportunities for graduate development, such as job shadowing, industry visits, and development assessment centres, would be impactful in achieving both higher levels of confidence and employability. It is interesting to note that both the USEM model and CareerEDGE model consider reflection/metacognition, self-efficacy, basic skills, and a level of work experience/understanding to be important to building employable graduates.

Furthermore, Jones (2015) presents a model of employability that is informed by one’s skills, confidence, and self-regulated learning. This model proposes that employability requires individuals who are confident and have the ability to manage their learning and skills. Jones (2015, p. 17) proposes a definition of employability as the “ability and attitude to apply and adapt knowledge and skills to current and future opportunities across a career path enabling contribution to a range of occupations in public, private, or not-for-profit sectors.” The underpinning concepts of this employability model display the need for knowledge and skills; the need for individuals with self-regulated learning abilities who are capable of adapting and broadening their knowledge; and the need for individuals to confidently apply one’s knowledge and ability (self-efficacy; Zimmerman, 1990; Bandura, 1995; Jones, 2015). This model proposes a virtues cycle between the skills that a person has obtained, their level of self-efficacy and self-regulated learning. The starting point of the process is largely unclear, but it seems logical that high levels of self-efficacy will enable self-regulated learning, which ultimately leads to greater efficacy in the workplace and employability. This cycle continues indefinitely through the graduate’s career, where bigger or more complex tasks require the acquisition of skills through self-regulated learning.

Another well-known employability model is conceptual model of graduate attributes of Bridgstock (2009) for employability that includes career management skills. This model proposes the importance of career management skills in achieving employability (i.e., variation of high-impact and long-term career capabilities), including one’s ability to intentionally self-manage and actively seek prospective opportunities (Bridgstock, 2009). Although having the relevant and desired skills is necessary in achieving employability, this model also sheds light on the underpinning traits and dispositions that are a critical component of employability. These underpinning traits and dispositions (e.g., sociability, taking initiative, willingness to learn, openness to experience, and self-efficacy) are known as the foundation of successfully developing and applying career management skills (Jarvis, 2003; McMahon et al., 2003; Bridgstock, 2009; Hoeller et al., 2014). Although self-efficacy is recognised as an underlying characteristic in achieving employability throughout various employability models, it proves to be a critical attribute to graduate employability success. Pinquart et al. (2003) agree by making mention of the importance of one’s motivation and self-efficacy, which play a pivotal role in a graduate’s transitional experience from the academic environment to the work environment.

The various theoretical employability models indicate that self-efficacy plays a rather important role in graduate employability, either as a direct effect or as mediator. A positive correlation between self-efficacy and employment search behaviour and graduate employment results has been found (Moynihan et al., 2003; Pinquart et al., 2003). Moreover, self-efficacy is also positively linked to academic achievement, motivation constructs, and self-controlled behaviour (such as awareness of learning methods utilised and time taken; Lowden et al., 2011; Morrison, 2014; Gharetepeh et al., 2015). Further studies have demonstrated that self-efficacy positively correlates with career adaptability (Öncel, 2014; Atitsogbe et al., 2019), as well as demonstrating higher levels of self-efficacy linked to higher levels of perceived employability (Ngo et al., 2017). Developing confident graduates is important for the preparation of facing unfamiliar challenges and opportunities that the ever-changing working world presents. Jones (2015) agrees by stating that those with high confidence levels are deemed as more employable due to their belief and ability to apply preferred behaviours and ways of work that lead to successful outcomes.

### Self-Efficacy

Self-efficacy forms the core mechanism for developing an individual’s motivation to exercise control over situations that affect their life (Sanchitra and Bandara, 2017). Self-esteem describes an individual’s belief regarding their ability to manage and carry out motivation, cognitive resources, and courses of action that will guide them to the success of specific tasks (Simons and Buitendach, 2013). This definition proposes that efficacy relates to the achievement of a precise task and is situation-specific. In addition to this, Bandura (1997) stated that the social-cognitive theory of self-efficacy is multi-faceted and varies across multiple circumstances and tasks.

Efficacy is not a trait, but rather a general capability that evolves over time and experience (Mazaheri and Yazdani, 2016). This belief is primarily shaped by four sources within self-efficacy, namely, mastery experience, vicarious experience, verbal persuasion, and physiological arousal. Allison and Keller (2004) discovered that a self-efficacy intervention involving all four self-efficacy development mechanisms led to advanced developments in older adult’s physical activity performance. Moreover, self-efficacy plays an important role not only in the work environment, but also in an individual’s everyday lifestyle (Lunenburg, 2011).

Studies have also shown that self-efficacy plays a mediational role in student’s selection of career choices. Studies of Pajares (2003) indicated that self-efficacy beliefs impact the choice of majors and career paths of tertiary students. Moreover, Pajares (2003) outlines those undergraduates tend to choose majors and career paths based on the fields they feel most proficient in and deter from those fields that they believe they are less proficient in or are less able to compete. Views of Bandura (1994) underscore this by stating that the higher an individual’s level of perceived self-efficacy, the wider the variety of career paths they seriously consider, the more interest they show in diverse career paths, and they are generally better prepared to deal with success and failures. Moreover, research shows that in relation to academic achievement, individuals with lower levels of self-efficacy achieve lower levels of academic success and continuous failure may lead to learned helplessness (Juan et al., 2018). Learned helplessness is a psychological state where an individual avoids tasks that require persistence, interpret failure as a result of their lack of skills, negatively perceive tasks as challenges, and display a lack commitment (Filippello et al., 2020).

Generalised self-efficacy is important for ongoing career success, but may also be very important during the application and recruitment process for graduates. While recruitment is a stage in achieving employment, many graduates experience the assessment and interviewing process for the first time, leaving them anxious and not knowing what to expect, impacting their confidence. Research on interviewing self-efficacy conducted by Tay et al. (2006) found that receiving feedback on interview performance influences an individual’s self-efficacy levels over time. This proves that job seekers with higher levels of self-efficacy in interview capabilities typically receive more work opportunities. Thus, the influence of higher levels of self-efficacy influences graduates’ success in the application process as well as their job success.

### Competency-Based Assessments

Competency-based assessments are popular in South Africa to assess managerial and graduate potential. It is believed that South Africa is the third largest user of assessment and development centres amongst 82 other countries internationally (Mulder and Taylor, 2015). The approach’s popularity in South Africa can be ascribed to a number of factors, but one of the biggest drivers is the gap in quality education and formal training between various racial groups. Standardised assessments, especially those related to the assessment of cognitive ability, often highlights the contrast and inequality in education quality, resulting in large group differences between racial groups (Laher and Cockcroft, 2013). In light of South Africa’s history, perceived fairness, and cultural appropriateness is very important. The use of psychological measures is also legally mandated by the Health Professions Act (Republic of South Africa, 1974) and the Employment Equity Act (Republic of South Africa, 1998), and their use is restricted if they are not able to demonstrate ethnic and gender fairness. Generally, competency-based assessments are regarded as fairer by participants due the high degree of fidelity between the simulations and real-world work situation (Thornton et al., 2015). Internationally, Competency Based Assessment also leads to smaller ethnic and gender group differences (Leong and Park, 2016). Additionally, competency-based assessments are strongly linked to current and future job performance (Al-Mannaee and Ryan, 2018).

As highly valued processes, assessment and development centres are actively utilised for purposes, such as recruitment, development, and retaining talent within organisations (Callow, 2010). These multi-purpose centres are known as a standardised process whereby multiple raters evaluate participant’s performance against pre-defined competencies which are assessed through a series of job-related simulations. These pre-defined competencies are related to the requirements and behaviours of a specific role. Typically, a development centre process usually occurs over a day or number of days where participants are asked to participate in and engage with a number of on-the-job simulations (Ballantyne and Povah, 2017).

Assessments may be conducted either by utilising the paper-pencil based technique or through electronic (simulated) assessment, thereby presenting participants with the opportunity to display their competencies in the given tasks (Mohamad et al., 2013). Assessment and development centres have many advantages, such as: (1) being able to measure complex characteristics, (2) seen as face valid and fair by those who participate in them, (3) has diminutive adverse impact, and (4) predicts a variety of criteria (Strudwick, 2017). The main difference between an assessment and development centre is that the former is utilised for selection purposes and the latter for personal and professional development purposes, which leads to organisational and team development (Sukalova and Hraskova, 2006).

Organisations benefit from development centres in aspects, such as (1) being seen as impartial and a robust approach to enhancing the employees’ and the organisations’ awareness of the individual’s skills, strength, and development areas, (2) by providing a unique opportunity to objectively observe and evaluate how employees execute tasks and activities, make decisions, relate to others, and exhibit self-awareness, and (3) acting as an effective tool for determining essential behaviours that are seen as imperative to employees’ current success and future potential (Sukalova and Hraskova, 2006). These same benefits may be beneficial in a classroom environment if development centres provide graduates with the opportunity to learn new skills, acting as a source of career preparation, boosting student confidence by developing self-awareness and illuminating blind spots, and acting as an objective resource to develop graduate attributes required by careers. The next section will look at the link between self-efficacy and development centres by framing the research problem and objectives in this study.

### The Link Between Self-Efficacy and Development Centres

Graduate self-efficacy is vital to academic and employability success since it is instrumental in overcoming obstacles, managing stressful situations, and achieving personal and professional goals. General research findings support this assumption, suggesting that the level of one’s self-efficacy has implications for changes in behaviour, stress management, and academic and career choices (Gharetepeh et al., 2015; Juan et al., 2018). On the other hand, those who have a weaker sense of self-efficacy have low ambitions and weaker commitment to goals, retreat from difficult tasks, dwell on adversity, are slow to gain confidence after experiencing failure, and easily encounter stress and depression (Mlatsheni, 2012).

The role of self-efficacy is important to graduate career and academic progression. Graduates typically face a myriad of stressors, and overcoming these professional, educational, and personal challenges is often the difference between success and failure. Bandura (1997) positions self-efficacy as a critical component in the amount of effort and perseverance applied to activities and tasks. This, in essence, speaks to an individual’s performance and persistence in achieving success in a specific task or situation. Numerous studies support the link between high levels of self-efficacy and scholastic achievement (Oriol-Granado et al., 2017).

Self-efficacy is influenced by an individual’s past experience of successes and failures, second-hand experience of the success and failure of others, developmental feedback, and the set of somatic-emotional reactions attached to performance beliefs. This touches on four sources of self-efficacy of Bandura (1997) discussed previously. Paton and Jackson (2002) states that development assessment centres act as a source of directly gained experience that incorporates behavioural modelling (which includes several participants), feedback, and opportunities to understand and develop methods of improvement that leads to improvement in self-efficacy, effort, and persistence. In this regard, Development Centres can initiate and activate the four processes that lead to higher levels of self-efficacy.

Studies conducted by Creed et al. (2001) found employability-enhancing interventions, like training and education, to positively lead to higher levels of confidence and esteem in unemployed individuals. Development centres also act as a source of training and a knowledge sharing tool. These centres provide a platform for graduates to acquire diverse skillsets, be equipped with desired graduate employability attributes and competencies, receive developmental feedback on ways to improve, and gain the confidence and belief to apply learnt abilities for prospective employment opportunities. While programmes and initiatives such as development centres are a useful tool in developing individuals, most of the time these opportunities are voluntary. Thus, graduate willingness to learn and ongoing growth mindset, are important attributes in achieving personal and professional success.

With the rapid and pervasive changes in the global, political, and economic systems, one of the key success factors is the ability of graduates to continuously learn and remain agile. Xing and Marwala (2017) concur by mentioning how globalisation, the fourth industrial revolution, the increasing demands for tertiary education, increasing competition, and collapsed geographical restrictions, have forced higher education into an extremely competitive environment where ongoing growth and education are critical for survival. To add to this pressure, graduates as new employees are expected to add value to organisations from the first day at work (Brits, 2018). This pressure to perform can have a profound impact on graduates and a high level of self-efficacy may be the key differentiator between initial success and failure.

This study proposes that development centres act as a useful intervention to improve graduate self-efficacy. The development centre is grounded in essential graduate employability attributes, appropriate assessment techniques, and ongoing development feedback that are applied in university settings. This application of development not only exposes graduates to real-life work simulations that improves their self-awareness and specific skills, but also creates a higher level of graduate self-efficacy. This sense of higher self-efficacy leads to the belief of successfully applying one’s gained knowledge and abilities to prospective career opportunities. Having the belief to apply one’s abilities (i.e., self-efficacy) is more significant than merely having the ability (Yorke, 2006).

### Research Objectives and Substantive Hypotheses

The primary objective of the study is to investigate the role of a development centre on generalised self-efficacy of graduate students. The secondary research objective is to investigate if the change in self-efficacy has a short-term or longer-term effect.

Based on the research objectives and the literature review, theorising suggests that graduate self-efficacy should increase over the short and long term once the graduates have gone through a development centre intervention. The following specific hypotheses guide the inquiry:

Hypotheses 1: A development centre intervention has a short-term effect on the generalised self-efficacy of graduate students.
Hypotheses 2: A development centre intervention has a longer-term effect on the generalised self-efficacy of graduate students.

## Materials and Methods

### Participants

Due to the nature of the study, a non-probability convenience sampling technique was used. The population for this study consisted of graduate students who were in the process of completing their graduate studies (Honours level) at a residential public university in South Africa. The population consisted of 95 postgraduate students. Out of the total number of 95 students, 17 graduate students volunteered to partake in the research project of which seven students were part of the intervention group, and 10 formed part of the control group. Table 1 represents the study sample characteristics for both the control group and the intervention group.

**Table 1:** Sample sociodemographic characteristics.

	Control group (*n*=10)		Intervention group (*n*=7)	
Variable	Frequency	Percentage	Frequency	Percentage
Gender				
Male	3	30.0%	4	57.1%
Female	7	70.0%	3	42.9%
Race				
Black/African	2	20.0%	3	42.9%
Coloured	8	80.0%	2	28.6%
Indian	0	0.0%	1	14.3%
White	0	0.0%	1	14.3%
Nationality				
South Africa	9	90.0%	6	85.7%
Zimbabwe	1	10.0%	1	14.3%
Marital status				
Married	2	20.0%	1	14.3%
Single	8	80.0%	6	85.7%
Home language				
English	6	60.0%	2	28.6%
Afrikaans	2	20.0%	2	28.6%
Shona	0	0.0%	1	14.3%
Swati	0	0.0%	1	14.3%
Zulu	0	0.0%	1	14.3%
Xhosa	1	10.0%	0	0.0%
Ikwerre	1	10.0%	0	0.0%
Work experience				
None	2	20.0%	1	14.3%
<6months	3	30.0%	2	28.6%
>12months	5	50.0%	4	57.1%

The information summarised in Table 1 suggests that most of the respondents were female in the control group (70%), Coloured (80%), single (80%), and English as primary home language (60%). In contrast, the intervention group was mostly more balanced with 57.1% males, Black (42.9%), single (85%), and with English as the primary home language (28.6%). Racial categorisation reported is aligned to the Employment Equity Act definitions (Republic of South Africa, 1998).

### Tools

In this particular study, two methods of data collection were utilised, namely questionnaires, and a development centre as the intervention. The development centre consisted of an in-basket assessment and a competency-based interview.

#### Generalised Self-Efficacy Questionnaire

In both the control and intervention group, the Generalised Self-Efficacy questionnaire (Schwarzer and Jerusalem, 1995) was administered before and after the development centre intervention. The general self-efficacy questionnaire is based on an individual’s general beliefs in their ability to respond to and manage environmental demands and challenges (Schwarzer, 2014). This instrument consists of a 10-item self-report questionnaire, scored on a four-point Likert Scale with 1 being “not at all true,” 2 being “hardly true,” 3 being “moderately true,” and 4 being “exactly true.” The instrument generally demonstrates strong internal consistency, and Cronbach alpha values ranging between 0.75 and 0.91 have been reported in applied studies (Scholz et al., 2002). In addition to this, the criterion-related validity of the instrument correlates positively with favourable emotions, optimisms, and work satisfaction (Schwarzer and Jerusalem, 1995). On the other hand, negative correlations were identified between generalised self-efficacy and depression, anxiety, burnout, stress, and health complaints (Schwarzer and Jerusalem, 1995). These findings provide support for the divergent and convergent validity of the measure. The measure is conceptualised to be uni-dimensional (Scholz et al., 2002) and includes statements such as “It is easy for me to stick to my aims and accomplish my goals,” “I can solve most problems if I invest the necessary effort,” and “When I am confronted with a problem, I can usually find several solutions.”

#### Development Centre Intervention

For this study, the development centre intervention included an in-basket assessment and a competency-based interview. An in-basket assessment is a tool and activity used to see how an applicant performs job-related duties within a given timeframe (Roberts, 2018). In-basket assessments require applicants to take action and structure a response of an employee in a hypothetical position on items, such as e-mails, memos, reports, records, and meeting minute requests (Schippmann et al., 1990). Moreover, competency-based interviews, also known as structured interviews, are interviews that have questions designed to elicit responses that allow the interviewers to measure the candidate against the competency profile developed for the position (Warech, 2002).

For the purpose of this study, only the intervention group participated in the development centre intervention. The intervention group completed a pre-test questionnaire (i.e., self-efficacy questionnaire), an in-basket assessment, a competency-based interview, and a post-test questionnaire (i.e., self-efficacy questionnaire) immediately after the development centre. The control group completed the generalised self-efficacy questionnaire prior to the start of the development centre intervention and 3months after the development centres.

The development centre was made up of six raters, who were trained on frame of reference training. Raters worked in pairs, with each pair assessing three candidates for the in-basket and competency-based interview. An observer guide was developed as preparation material for each rater. This guide contained the assessment competency guide, assessment material, observer programme, and rating sheets. All of the raters were graduate students in the process of completing the final year of their Masters coursework. As part of a module in Advanced Assessment (BPS 820) they received extensive training on the principles of observing, recording, classifying, and evaluating behaviour against the competency framework. All the raters were coloured and female.

The in-basket and competency-based interview assessments were developed to assess 10 main competencies. These competencies are relevant knowledge, planning and organising, oral and written communication, action orientation, ability to learn, attention to detail, analytical thinking, adaptability, and initiative. The competencies were derived by reviewing various job descriptions of an Industrial Psychology entry level professional. Moreover, Industrial Psychologists across various organisations were consulted on their perspective of core competencies that graduate Industrial Psychologists require. This was then cross referenced and analysed against universal competencies in order to derive the final competencies. These competencies with associated definitions can be viewed in Table 2.

**Table 2:** Competency grid.

Competencies	Assessments	
	In-basket activity	Competency-based interview
Relevant knowledge and skill *Possessing the necessary knowledge and skills to meet the job demands*.	X	X
Planning and organising *Organising information and determining courses of action for oneself and others, taking relevant factors into account*.	X	X
Communication – Oral *Effective two-way communication with others, including verbal and gestural expression, and listening*.	–	X
Communication – Written *Clear written expression of ideas or information*.	X	–
Action Orientation *Willingness to take action to accomplish tasks, maintaining a high level of motivation and energy*.	X	X
Ability to learn *Ability to assimilate, understand, and apply new information*.	–	X
Attention to detail *Taking relevant and complex details into account*.	X	X
Analytical thinking *Understanding a situation by breaking it apart into smaller pieces or tracking the implications of situations in a step by step way*.	X	X
Adaptability *Maintaining effectiveness in varying environments and with different people, tasks, and responsibilities*.	X	X
Initiative *Originating action and taking the initiative without having to be prompted*.	X	X

The assessments were rated on a five-point Likert rating scale. The competencies and respective behavioural indicators were utilised as a guide when rating participants, while the rater utilised a Behavioural Observation Scale (BOS) approach. The five-point Likert scale rated 1, being well below requirements or no evidence of behavioural indicator or response; 2, being below requirements; 3, being meets most requirements with development; 4, being above requirements, and lastly 5, being well above requirements or answers all behavioural indicators and does much more than required.

Once all simulations had been conducted, the raters were required to go through a data-integration session where participants’ scores were discussed, and scoring was finalised. The data-integration session was where the raters came to a consensus around participant scores by comparing participant results that were aligned to the agreed competencies and behaviours. Thereafter, participant development reports were written, password protected, and sent *via* email communication directly to the researcher and supervisor to send out to the respective participants.

### Procedure

The research study was presented to the study population and students could volunteer to take part. The group of graduates that were available to take part in the development centre on the advertised date was assigned to the intervention group, while applicants that were not available were considered for the control group. The development centre was conducted in a centralised venue on campus due to ease of access for all participants. On the day of the intervention, the intervention group was required to complete the informed consent form, self-efficacy questionnaire (pre-test), a demographic questionnaire, go through the development centre programme (i.e., in-basket assessment and a competency-based questionnaire), and then complete the self-efficacy questionnaire again. The control group was required to complete the informed consent form, self-efficacy questionnaire (pre-test), and a demographic questionnaire only. Three months later both intervention and control groups were given a post-test self-efficacy questionnaire to complete. Once the intervention concluded, each participant received an email thanking them for their participation in the study, while the intervention group additionally received an assessment report for their participation in the development centre. In addition to the written report, verbal feedback was provided for the candidates that participated in the development centre. The candidates that were allocated to the control group were given the opportunity to participate in the annual development centre that would take place at the same time 1year later. None of the respondents in the control group opted in to participate in the development centre the following year.

After all data were received, the researcher reviewed, analysed, and contextualised the data through excel and SPSS (IBM Corp, 2015).

### Statistical Analysis

As the design of this study involves assessing self-efficacy across both time (pre- and post- assessment center intervention) and cohorts (treatment and control), analysis was challenged by the potential for baseline differences in treatment and control group self-efficacy levels. This presents an additional source of error when attempting to compare changes within cohorts, between cohorts. Further, small sample have low statistical power to detect type II errors, which was a concern in the current study.

Typically, inter-group differences (whether groups are independent or dependent) are assessed by parametric test (equivalence testing in SEM or Independet *t*-test). Because of our small sample, this test will not be possible, as the *t*-test assumes certain distribution characteristics including multivariate normal distribution of continues scores (Krieg, 2020). The Mann-Whitney U Test is among the preferred non-parametric alternatives to the *t*-test, and was selected because the approach overcomes some of the stringent data requirements of parametric test (Fay and Proschan, 2010). Further, the ranking system utilised by the Mann-Whitney U test effectively standardizes the distributions under analysis (Fay and Proschan, 2010), limiting any concern over error produced by baseline inter-group self-efficacy differences.

In order to evaluate the differences across time, the Friedman (1937) test was utilised. The Friedman test is a non-parametric alternative to one-way repeated ANOVA. Significant results on the Friedman Test suggest that intragroup differences are detected over time (Krieg, 2020). The Friedman Test was followed up with Wilcoxon Signed Rank test to test for *post-hoc* differences across time. In the current investigation, time 1 was considered the baseline reading and was used to compare changes at time 2 and 3.

### Ethical Considerations

Anonymity and confidentiality were taken into account in the informed consent form by explaining the purpose of the study, researcher details, that the participant will remain anonymous, that their responses in the study will remain confidential, that participation is voluntary, and any personal identity will not be shared with any third parties or vendors. This study has also undergone ethical clearance through the University’s ethics committee.

## Results

The primary goal of the study was to investigate the following hypotheses:

Hypotheses 1: A development centre intervention has a short-term effect on the generalised self-efficacy of graduate students.
Hypotheses 2: A development centre intervention has a long-term effect on the generalised self-efficacy of graduate students.

In order to test these hypotheses, a within-between subject research design was utilised. The first step was to test if there were any significant intergroup differences at time 1 between the intervention and control group. This was followed up by testing for difference between the group at time point 3s. This was done by means of the Mann-Whitney U test, a non-parametric test, due to the small sample sizes of the control and intervention groups.

The “within” part of the research design was carried out using the Friedman Test followed by the Wilcoxon signed rank test. In each of the analyses, time 1 (pre-intervention) was used as the baseline measure. In other words, the immediate (i.e., short term) effect was assessed by comparing time 1 with 2, and the long term effect was tested by comparing time 1 with time 3. If the Wilcoxon signed ranks indicate a significant change between time 1 and time 2, this change can be considered a significant immediate effect. If the change between time 1 and 3 is significant, it is considered a significant long-term effect.

### Tests for Mean Differences in Self-Efficacy Between the Intervention and Control Group (Mann-Whitney U Test)

The information in Tables 3, 4 suggest that that for time 1 (SE_T1), the Mann-Whitney U Test revealed that there was no statistically significant difference in the mean scores in the graduate’s self-efficacy levels for the intervention group (Mean Rank=9.14, *N*=7) compared to the control group (Mean Rank=8.90, *N*=10); (*U*=34,000, *z*=−0,098, *p*=0.922, *r*=−0.00033).

**Table 3:** Ranks table.

Group		*N*	Mean rank	Mean values	Sum of ranks
SE_T1 FACTOR SCORE SE	1 INTERVENTION	**7**	**9,14**	**3,4429**	64,00
	2 CONTROL	**10**	**8,90**	**3,4100**	89,00
	Total	17			
SE_T3 FACTOR SCORE SE	1 INTERVENTION	**7**	**10,50**	**3,6667**	63,00
	2 CONTROL	**10**	**7,30**	**3,4300**	73,00
	Total	17			

**Table 4:** Test statistics table.

	SE_T1 FACTOR SCORE SE	SE_T3 FACTOR SCORE SE
Mann-Whitney U	34,000	18,000
Wilcoxon W	89,000	73,000
*Z*	**-0,098**	**-1,319**
Asymp. Sig. (two-tailed)	**0,922**	**0,187**
Exact Sig. [2*(one-tailed Sig.)]	0.962^b^	0.220^b^

b *Not corrected for ties*.

The mean rank values for the control and intervention groups are displayed in Table 3. The significance test between groups at time 1 and 3 is displayed in Table 4 below. Table 4 suggests that there is no statistically significant difference in the graduate’s self-efficacy levels for the intervention group (Mean Rank=10.50, *N*=6) and the control group (Mean Rank=7.30, *N*=10); (*U*=18,000, *z*=−1,319, *p*=0.187, *r*=−0.00456).

The results from the foregoing analyses suggests that there were no statistically significant differences between the control and intervention group on generalised self-efficacy at time 1 and time 3.

#### Tests for Mean Differences in Self-Efficacy Over Time (Friedman Test)

The Friedman test is the non-parametric test which is a non-parametric alternative to the one-way ANOVA with repeated measures. This test is utilised to test for differences between groups when the dependent variable being measured is ordinal (Laerd Statistics, 2018).

The mean ranks and significant tests for the intervention group are presented in Tables 5, 6. The test statistics in Table 6 indicated that there was not a statistically significant difference in the intervention group’s self-efficacy levels across time 1 (SE_1) which is the pre-intervention, time 2 (SE_2) which is the post-intervention, and time 3 (SE_3) which is the post 3month follow up. This is indicated by a non-significant value of *p* of 0.247. Comparing the Mean Ranks across time 1, time 2, and time 3 shows that there was a practical significant increase in the intervention groups self-efficacy levels over time. From time 1 to 2, there was a 5.8% increase in the mean value and 7.6% increase between time 1 and 3. However, due to the relatively small sample size, this increase in Self Efficacy over time was not statistically significant (Table 7).

**Table 5:** Ranks.

Group		Mean Rank	
1 INTERVENTION	SE_T1 FACTOR SCORE SE	1.50	SE_T2 FACTOR SCORE SE	2.33	SE_T3 FACTOR SCORE SE
	2.17

**Table 6:** Test statistics.

1 INTERVENTION	*N*	6
	Chi-Square	2,800
	Df	2
	Asymp. Sig.	**0,247**

**Table 7:** Intervention group self-efficacy over Time 1, 2, and 3.

Group		Mean	SD	*N*
1 INTERVENTION	SE_T1 FACTOR SCORE SE	**3,4333**	0,39328	6
	SE_T2 FACTOR SCORE SE	**3,6000**	0,34641	6
	SE_T3 FACTOR SCORE SE	**3,6667**	0,29439	6

**Figure 1:**
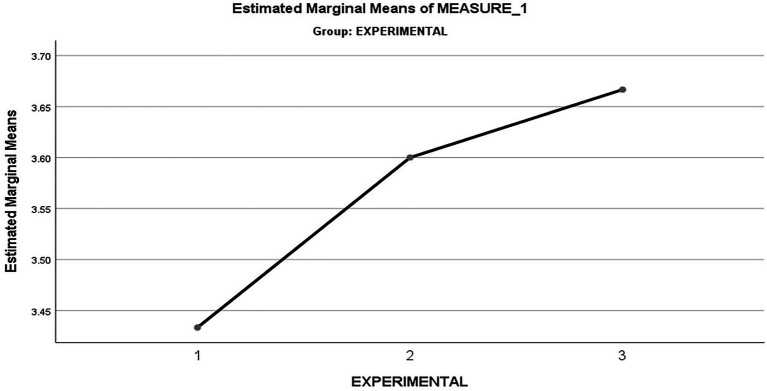
Intervention Group Self-efficacy over Time 1, 2, and 3.

Figure 1 provides a graphical view of the increase in mean values from time 1 to 3 for the Intervention Group. Intervention Group Self-efficacy over Time 1, 2, and 3, displays a graphical view of the immediate change/increase in the intervention groups self-efficacy between time 1 and 2 (time 1 mean=3,4,333; time 2 mean=3,6,000). From time 2 to 3, the level of self-efficacy gradually increases/stabilizes (time 2 mean=3,6000; time 3 mean=3,6667).

#### *Post-hoc* Tests for Repeated Measurements in Self-Efficacy (Wilcoxon Signed Rank Test)

The Wilcoxon Signed Rank Test is a statistical evaluation of the mean of two dependent groups. This non-parametric test works with metric data (interval or ratio) that is not multivariate normal, or with ranked/ordinal data (Statistics Solutions, 2019).

In this study, the Wilcoxon Signed Rank test was utilised to measure whether there was statistical significant differences within each group between time 1 and 2 as well as between time 1 and 3. In both, the paired comparisons time 1 was used as the baseline measure reflect changes over time.

Results in Tables 8, 9 indicate that there was statistically significant differences between the mean ranks for the intervention group between time 1 and 2 (*z*=−2,041, *p*=0.041) but not between time 1 and time 3 (*z*=−0.171, *p*=0.865). These results suggest that the intervention had an immediate effect on generalised self-efficacy but not a longer term effect for the intervention group.

**Table 8:** Intervention and Control Descriptive Statistics.

Group		*N*	Mean	SD	Minimum	Maximum
1 INTERVENTION	SE_T1 FACTOR SCORE SE	7	**3,4429**	0,35989	2,90	4,00
	SE_T2 FACTOR SCORE SE	7	**3,6000**	0,31623	3,10	4,00
	SE_T3 FACTOR SCORE SE	7	**3,6667**	0,29439	3,30	3,90
2 CONTROL	SE_T1 FACTOR SCORE SE	10	**3,4100**	0,44083	2,70	3,90
	SE_T2 FACTOR SCORE SE	0				
	SE_T3 FACTOR SCORE SE	10	**3,4300**	0,38601	2,90	3,90

**Table 9:** Intervention and control test statistics.

Group		SE_T2 FACTOR SCORE SE – SE_T1 FACTOR SCORE SE	SE_T3 FACTOR SCORE SE – SE_T1 FACTOR SCORE SE
1 INTERVENTION	*Z*	–2.041^b^	–1.261^b^
	Asymp. Sig. (two-tailed)	**0,041**	0,207
2 CONTROL	*Z*		–.171^b^
	Asymp. Sig. (two-tailed)		**0,865**

b *Based on negative ranks*.

**Figure 2:**
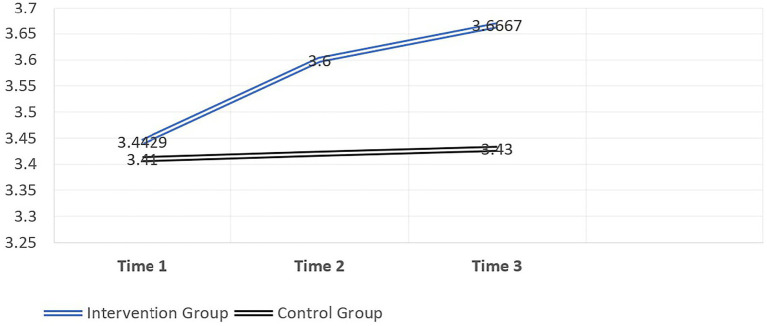
Intervention and Control Mean over Time 1, 2, and 3.

Moreover, Tables 8, 9 indicate that there was no statistically significant difference between the mean ranks for the control group between time 1 and 3. This suggests that there was no longer term effect visible for the control group. The difference over time in self-efficacy scores can be seen graphically in Figure 2, where it is evident that for the control group there is relatively little increase in the generalised levels of self-efficacy. However, for the intervention group, there is a relatively strong increase in self-efficacy after the development centre and after 3months (albeit not statistically significant).

The results largely supported the main research hypothesis that an assessment centre intervention is successful in bringing about a short- and longer term change in generalised self-efficacy. Although the results were not statistically significant, the diverging lines in Figure 2 largely indicate that the intervention had a pronounced impact on the intervention group when compared to the control group.

## Discussion

### Background

The aim of the current study was to investigate if development centres can improve the generalised self-efficacy of graduates over the short and longer term. The primary research question was centred on the idea that most graduates struggle to bridge the gap between the theoretical and practical settings. Bridging the gap between tertiary studies and the workplace is critical for the business sector as well as the Higher Education Institutions. Universities aim to produce graduates that can make a material impact in the applied context, while organisations aim to attract the best talent coming out of universities. To this end, the gap between theory and practice remains an inconvenience for organisations and institutions of higher learning.

### Discussion of the Findings

The results indicated that the development centre had a practically significant impact on the self-efficacy levels of the intervention group. Moreover, results of the statistical analysis suggest a strong continuous improvement in the generalised self-efficacy of the intervention group over a 3-month period. On the other hand, the self-efficacy levels of the control group had not increased nor decreased throughout the study and stayed fairly stable over the 3-month period. Although the results were not statistically significant, the research shows that the intervention had a definite impact on the intervention group, relative to the control group. This provides promising, though not statistically significant, evidence that development centres have an impact on graduates’ generalised self-efficacy levels over the short and long-term. The results are discussed in more detail in the section below.

#### Hypotheses 1

Hypotheses 1 tested whether the development centre intervention has a short-term effect on generalised self-efficacy levels of graduate students. The results supported this hypothesis, indicating that the development centre – as an intervention – has an immediate effect on graduate self-efficacy. This comparison was statistically significant, despite the relatively small sample size. The intervention group displayed an immediate positive change in self-efficacy after participating in the development centre. The results seem to suggest that implementing development centres interventions into tertiary higher education institutions and its curriculum can increase perceived generalised self-efficacy in the short term. This study proved that it can benefit graduates by increasing their level of self-insight and self-efficacy levels. Other studies have found development centres to impact participants’ self-awareness through performance feedback, outlining essential behaviours critical to their success, and improving graduate academic performance and employability (Sukalova and Hraskova, 2006; Van Wyk and du Toit, 2018).

While assessment interventions pose many benefits, assessment feedback plays an integral part in a student’s learning process (Hill and West, 2020). In addition, assessment approaches equip graduates in experiencing real-world training simulations (see, e.g., Stevens et al., 2017). It is evident that development assessment interventions holistically benefit students by improving their technical and interpersonal skills, creating self-awareness, identifying areas of strength and development through feedback and awareness, and preparing them for the world of work through live simulations.

#### Hypotheses 2

Hypotheses 2 tested whether the development centre intervention has a longer-term effect on generalised self-efficacy levels of graduate students. The results indicate the longer-term effect was not statistically significant, but practical significance indicated there was a consistent increase in self-efficacy from time 1 (pre-test) to time 3 (post 3-month test) for the intervention group. Thus, across all three measurement points, there was an increase in generalised self-efficacy for the intervention group, but not for the control group. This indicates that the development centre had a promising impact on the short-term and long-term self-efficacy of graduate students.

The results from the study suggest that development centres can play a pivotal role in individual growth and performance of graduates, especially when it comes to self-efficacy. These centres help identify individual strengths and areas of development, determine essential behaviours for current success and future potential, and provide participants with feedback on their performance (Sukalova and Hraskova, 2006; Van Wyk and du Toit, 2018).

While development centres have always been used very effectively in the world of work, Willis (2007, p.32) and Earl (2014) state that these assessments should be assimilated into lectures and classrooms, by providing distinctive links to personal and professional outcomes. One way of achieving this goal would be for universities to start incorporating elements of assessment centres into their teaching pedagogy and curriculum. Moolman (2017) states that it is vital that tertiary institutions constantly build key job requirements into development centre designs to make sure graduates get exposure to on-the-job tasks and activities. When tertiary institutions align their curriculum to the workplace, graduates will integrate into the workforce seamlessly and make an immediate impact in the organisation and wider economy. Managing director of ManpowerGroup South Africa, Lyndy van den Barselaar, posits that investing in the advancement and upkeep of career service centres should be a top priority in South Africa as these centres assist in closing the skill gaps and assist organisations to select the best talent from tertiary training institutions (The Skills Portal, 2018). While investing in development centres may be costly, tertiary institutions should find alternative methods to integrate development centre aspects into their curriculum. Practical examples include providing practical classes, focus groups, online chat groups, career counselling, assessment, and development feedback that aims to enhance graduate employability and self-efficacy.

Although various research studies have looked into graduate employability and development centres, limited research has focused on developing graduate self-efficacy. Employability is when graduates obtain and maintain employment with the appropriate skills and qualities, while continuously developing personally and professionally (Hillage and Pollard, 1998; Bridgstock, 2009). On the other hand, self-efficacy is a broad competency that may help graduates to develop the skills that is needed to stay employable. Recent literature suggests that efficacy is not a trait, but a general capability that evolves over time and experience (Mazaheri and Yazdani, 2016). This tells us that self-efficacy needs to be developed and maintained in order to have a positive impact in one’s ability to accomplish tasks and overcome challenges. Moreover, multiple research studies have found self-efficacy as an important graduate attribute across numerous fields of study (Harvey, 2000; Lowden et al., 2011; Morrison, 2014; Gharetepeh et al., 2015). When graduates have a strong sense of self-efficacy, they are able to approach difficult tasks, set stimulating goals and experience lowered levels of stress, depression, and anxiety (Bandura, 1994). Thus, while the right graduate attributes and skills are important to academic and work success, the level of the graduates’ self-efficacy in turn determines this success.

Research concurs by stating that an individual’s environment is to an extent affected by their judgements of their own abilities (Aghdami Baher et al., 2009). However, despite both tertiary institutions and organisations placing great value on graduate self-efficacy, or at least it is proposed benefits; there has been a lack of effort to develop this attribute. Development centres are one way to have a positive impact on self-efficacy, which assist in gaining insight into the relative strengths and weaknesses of graduates who may be applying for jobs. While development centres focus on improving certain competencies of individuals for specific positions, universities should utilise these centres to develop graduate attributes which in turn develops graduate confidence to overcome future workplace challenges. Developing confident graduates may prove the single most important determinant of graduate success in the workplace. Moreover, it also influences the degree of their effort and perseverance when learning challenging tasks (Lunenburg, 2011). As this study has suggested, development centres positively influence graduates’ self-efficacy over the short and medium term. This trend was not found with regards to the control group. This research informs us that development centres have certain additional benefits other than gaining diagnostic information about graduates that may be beneficial for graduates as well as organisations.

### Limitations

The major limitation of the study is the relatively small size of the control and intervention groups. Although the statistical analysis suggested that the development centre had an influence on the self-efficacy perceptions of the intervention group, these inferences should be interpreted with caution due to the small sample size. More data need to be collected in future to confirm the pattern of results. Another potential limitation of the study may be the lack of control variables. For example, it cannot be ruled out that the two groups were similar in terms of achievement motivation. It is well known that achievement motivation can have an impact on test performance which in turn will have an impact on general self-efficacy. Achievement motivation is an internal driving force that is said to impact how an individual performs and achieves a task or expresses an accomplishment (Bao and Zhou, 2017). However, Shokhmgar et al. (2018) state that self-efficacy is in fact a determining element for achievement motivation. Based on various research studies, achievement motivation and self-efficacy seem to correlate when it comes to academic achievement and work performance (Bao and Zhou, 2017; Saeid and Eslaminejad, 2017; Benawa, 2018; Shokhmgar et al., 2018; Saadat et al., 2019).

Another limitation was the raters’ level of experience in evaluating participants. Although the raters had gone through extensive frame of reference training with an experienced instructor, for most raters, this was their first time scoring competency-based simulations. Although we acknowledge that the lack of experience may limit their ability to observe, record, classify, and evaluate behaviour, the benefit may be that these raters do not have ingrained bias in their rating process. In a study by Leckie and Baird (2011), it was found that the ratings between experienced and new raters did not on average deviate significantly. Previous research however indicates that experienced raters considered factors that were not in the scoring rubric and this can introduce bias in the ratings. More inexperienced raters tend to depend on the scoring criteria more closely (Cumming, 1990).

Moreover, the study only included data from a single university and a single class of graduate students in the same discipline. Although one would expect the results from the current study to extend to other settings and disciplines, this is an assumption that should best be tested.

Finally, the results on self-efficacy were collected by means of self-report instruments. Self-report measures typically suffer from impression management and faking behaviour – it would be more ideal to consider alternative data collection methods, including observations and online assessments that have validation tests for authentication reasons.

### Recommendations and Considerations for Future Research

Based on the findings and discussion, it is evident that development centres have a meaningful and positive impact on increasing graduate self-efficacy. Recommendations to implement development centres at universities should become an essential part of a graduates’ journey. It is important to embed some elements of development centres in the classroom experience and assessments to build self-efficacy from the 1st-year level. Problem based learning has been extensively used in universities to promote self-development, mastery and self-efficacy (Demirören et al., 2016; Masitoh and Fitriyani, 2018). Problem based learning is a method of instruction where students learn through a pedagogical process with the focus on problem-solving (Smith and Hung, 2017). This instructional learning approach enhances the students’ motivation by obtaining knowledge and assimilating it by utilising practical problem solving (Wijnia et al., 2011). It is clear that this method of learning contains various design elements that overlap with the development centre approach.

The objective of this study was to ascertain whether development centres develop confident graduate students over the short and long term. However, this study has not thoroughly investigated the mechanisms of how graduate confidence levels increases. Here, the opportunity for further research can be to unpack the means and mechanisms of developed self-efficacy through development centre initiatives. Similarly, control variables such as levels of motivation and work experience should be considered.

Moreover, the consideration of different assessment strategies and methods could be reviewed for future research. This study focused on in-basket assessments and competency-based interviews that had specific competencies related to Industrial Organisational Psychology students. Alternative assessments that are grounded in graduate attributes relevant to the working world should be considered, for example, leaderless groups, case studies, focus groups, and role plays.

In addition to the above, the impact of development centre interventions in graduate careers and work experience should be considered. Graduates who undergo extensive engagement with development centre interventions could be studied. The questions of whether the engagement has resulted in an easier transition from university setting to the working world, whether it positively assisted in career progression, made them more employable, and helped them to develop the essential attributes organisations seek are all aspects that could be investigated.

### Conclusion

The objective of this research study was to investigate whether development centres can be used to gain diagnostic information on graduates and improve their general levels of self-efficacy. This study revealed that implementing development centres within universities lead to a positive increase in the generalised self-efficacy levels of graduates. In addition, the findings revealed that development centres have an immediate impact on generalised levels of self-efficacy, which is maintained over the long-term. Graduate development is essential in building confident individuals to take on various new challenges that the world of work presents. Confident graduates are ultimately more courageous in taking risks and making better decisions, are able to tackle challenges better, set stimulating goals and commit to them, experience lower levels of stress, anxiety, and depression, and put more effort and perseverance into learning challenging tasks. It is clear from the research that developing graduates with a high level of self-efficacy is critical, as the graduates we develop today are the graduates who impact our future.

## Data Availability Statement

The raw data supporting the conclusions of this article will be made available by the authors, without undue reservation.

## Ethics Statement

The studies involving human participants were reviewed and approved by Humanities and Social Science Research Ethics Committee of the University of the Western Cape – H18/6/26. The patients/participants provided their written informed consent to participate in this study.

## Author Contributions

MW, JB, and MP contributed equally to the MS and worked on the discussion section. The core of the MS was based on the unpublished thesis of MW. JB did the data analyses and integration while MP formulated the literature review section framing of hypotheses. All correspondence should be directed to JB. All authors contributed to the article and approved the submitted version.

## Conflict of Interest

The authors declare that the research was conducted in the absence of any commercial or financial relationships that could be construed as a potential conflict of interest.

## Publisher’s Note

All claims expressed in this article are solely those of the authors and do not necessarily represent those of their affiliated organizations, or those of the publisher, the editors and the reviewers. Any product that may be evaluated in this article, or claim that may be made by its manufacturer, is not guaranteed or endorsed by the publisher.

## References

[ref1] Aghdami Baher A. Najjarpoor Ostadi S. Livarjani S. H. (2009). Relationship between emotional intelligence and sense of self-efficacy and burnout among staff of Islamic Azad University of Tabriz. J. Educ. Sci. 2, 99–119. doi: 10.17795/whb-30445

[ref2] Allison M. J. Keller C. (2004). Self-efficacy intervention effects on physical activity in older adults. West. J. Nurs. Res. 26, 31–46. doi: 10.1177/0193945903259350, PMID: 14984643

[ref3] Al-Mannaee N. S. Ryan J. C. (2018). Examining a competency model of workplace learning: An assessment of participants’ reactions. Int. J. Work Organ. Emot. 9, 107–124. doi: 10.1504/IJWOE.2018.10012448

[ref4] Atitsogbe K. A. Mama N. P. Sovet L. Pair P. Rossier J. (2019). Perceived employability and entrepreneurial intentions across university students and job seekers in Togo: The effect of career adaptability and self-efficacy. Front. Psychol. 10:180. doi: 10.3389/fpsyg.2019.00180, PMID: 30800087 PMC6376950

[ref5] Awan W. A. Ameen K. (2020). What do business employers want? A sequential mixed methods exploration of information professionals’ competencies. Global Knowledge Memory Commun. 69, 665–680. doi: 10.1108/GKMC-03-2020-0029

[ref6] Ballantyne I. Povah N. (2017). Assessment and development centres. London: Routledge.

[ref7] Bandura A. (1994). “Self-efficacy,” in Encyclopedia of Human Behavior, Vol. 4 (New York: Academic Press), 71–81.

[ref8] Bandura A. (1995). Self-Efficacy in Changing Societies. New York: Cambridge University Press.

[ref9] Bandura A. (1997). Self-Efficacy: The Exercise of Control. New York: Freeman.

[ref10] Bao J. Zhou X. (2017). Entrepreneurial achievement motivation, self-efficacy and strategic change: A multiple mediation analysis. Revista de la Facultad de Ingeniería 32, 570–577.

[ref11] Benawa A. (2018). The important to growing self-efficacy to improve achievement motivation. IOP Conf. Ser. Earth Environ. Sci. 126:012086. doi: 10.1088/1755-1315/126/1/012086

[ref12] Bennett D. Ananthram S. (2021). Development, validation and deployment of the employability scale. Stud. High. Educ. 1-15. doi: 10.1080/03075079.2021.1888079

[ref13] Bridgstock R. (2009). The graduate attributes we’ve overlooked: enhancing graduate employability through career management skills. High. Educ. Res. Dev. 28, 31–44. doi: 10.1080/07294360802444347

[ref14] Brits H. J. (2018). Assessing employer satisfaction: An attempt to enhance graduate employability at an institution of higher learning. South Afr. J. Higher Educ. 32, 39–53. doi: 10.20853/32-5-2571

[ref15] Callow T. (2010). Assessment and development: Centre stage. Employment Today. 28–31. Available at: https://psytech.com/Content/Media/employment_today_assessment_and_development_centres_feb_2010.pdf (Accessed April 05, 2019).

[ref16] Creed P. A. Bloxsome T. D. Johnston K. (2001). Self-esteem and self-efficacy outcomes for unemployed individuals attending occupational skills training programs. Community Work Fam. 4, 285–303. doi: 10.1080/01405110120089350

[ref17] Cumming A. (1990). Expertise in evaluating second language compositions. Lang. Test. 7, 31–51. doi: 10.1177/026553229000700104

[ref18] Dacre Pool L. Sewell P. (2007). The key to employability: developing a practical model of graduate employability. Educ. Train. 49, 277–289. doi: 10.1108/00400910710754435

[ref19] Demirören M. Turan S. Öztuna D. (2016). Medical students’ self-efficacy in problem-based learning and its relationship with self-regulated learning. J. Med. Educ. 21:30049. doi: 10.3402/meo.v21.30049, PMID: 26987386 PMC4796725

[ref20] Du Preez M. van der Merwe L. J. Swart S. B. (2019). Employability skills: what is required of consumer sciences graduates? J. Consum. Sci. 47, 92–104.

[ref21] Earl L. M. (2014). “Looking forward: evaluation in New Zealand education,” in A Developmental and Negotiated Approach to School Self-Evaluation (Advances in Program Evaluation), Vol. 14. (Bingley: Emerald Group Publishing Limited), 179–193.

[ref22] Fay M. P. Proschan M. A. (2010). Willcox-Mann-Whitney or t-test? On assumptions for hypothesis tests and multiple interpretations of decision rules. Stat. Surv. 4, 1–39. doi: 10.1214/09-SS051, PMID: 20414472 PMC2857732

[ref23] Filippello P. Buzzai C. Costa S. Orecchio S. Sorrenti L. (2020). Teaching style and academic achievement: The mediating role of learned helplessness and mastery orientation. Psychol. Sch. 57, 5–16. doi: 10.1002/pits.22315 33136418

[ref24] Freudenberg B. Cameron C. Brimble M. (2010). The importance of self: developing students’ self-efficacy through work integrated learning. Int. J. Learn. 17, 479–496. doi: 10.18848/1447-9494/CGP/v17i10/58816

[ref25] Friedman M. (1937). The use of ranks to avoid the assumption of normality implicit in the analysis of variance. J. Am. Stat. Assoc. 32, 675–701. doi: 10.1080/01621459.1937.10503522

[ref26] Gharetepeh A. Safari Y. Pashaei T. Razaei M. Kajbaf M. B. (2015). Emotional intelligence as a predictor of self-efficacy among students with different levels of academic achievement at Kermanshah university of medical sciences. J. Adv. Med. Educ. Prof. 3, 50–55. PMID: 25927067 PMC4403564

[ref27] Hamilton M. Carbone A. Gonsalvez C. Jollands M. (2015). “Breakfast with ICT employers: What do they want to see in our graduates” in *Proceedings of the 17th Australasian Computing Education Conference*; January 27-30, 2015. Available at: https://50years.acs.org.au/content/dam/acs/50-years/journals/crpit/Vol160.pdf#page=45 (Accessed November 16, 2021).

[ref28] Harvey L. (2000). New realities: the relationship between higher education and employment. Tert. Educ. Manag. 6, 3–17. doi: 10.1023/A:1009685205201

[ref29] Hill J. West H. (2020). Improving the student learning experience through dialogic feed-forward assessment. Assess. Eval. High. Educ. 45, 82–97. doi: 10.1080/02602938.2019.1608908

[ref30] Hillage J. Pollard E. (1998). Employability: Developing a Framework for Policy Analysis. London: DfEE.

[ref31] Hoeller P. Joumard I. Koske I. (2014). Reducing income inequality while boosting economic growth: can it be done? Evidence from OECD countries. The Singapore Economic Review 59, 181–202. doi: 10.1142/S0217590814500015

[ref32] Humburg M. Van der Velden R. Verhagen A. (2013). The Employability of Higher Education Graduates: The employer’s Perspective. Maastricht, The Netherlands: European Commission, 1–131.

[ref33] IBM Corp (2015). IBM SPSS Statistics for Windows. Version 23. Armonk: NY.

[ref34] Jacobs R. R. Griswold K. R. Swigart K. L. Loviscky G. E. Heinen R. L. (2018). From campus to corporation: using developmental assessment centers to facilitate students’ next career steps. J. Nat.Colleg. Honor. Council 567, 125–154.

[ref35] Jarvis P. (2003). Career management skills: Keys to a great career and a great life. National Life/Work Centre. 1–8. Available at: www.crccanada.org/symposium (Accessed January 14, 2020).

[ref36] Jones C. E. (2015). Does studying postgraduate management education increase students’ perceptions of their employability? PhD Thesis. (Birmingham, England: Aston University). Available at: http://publications.aston.ac.uk/id/eprint/30861/ (Accessed November 16, 2021).

[ref37] Juan A. Hannan S. Namome C. (2018). I believe I can do science: self-efficacy and science achievement of grade 9 students in South Africa. S. Afr. J. Sci. 114, 1–7. doi: 10.17159/sajs.2018/20170269

[ref38] Knight P. T. Yorke M. (2002). Employability through the curriculum. Tert. Educ. Manag. 8, 261–276. doi: 10.1080/13583883.2002.9967084

[ref39] Krieg E. J. (2020). Statistics and Data Analysis for Social Science 2nd *Edn.* Thousand Oaks, CA: SAGE Publications.

[ref40] Laerd Statistics (2018). Friedman test in SPSS statistics. Available at: https://statistics.laerd.com/spss-tutorials/friedman-test-using-spss-statistics.php (Accessed November 13, 2020).

[ref41] Laher S. Cockcroft K. (2013). “Contextualising psychological assessment in South Africa,” in Psychological Assessment in South Africa: Research and Applications. eds. Laher S. Cockroft K. (Wits University Press), 1–16.

[ref42] Leckie G. Baird J.-A. (2011). Rater effects on essay scoring: A multilevel analysis of severity drift, central tendency, and Rater experience. J. Educ. Meas. 48, 399–418. doi: 10.1111/j.1745-3984.2011.00152.x

[ref43] Leong F. T. L. Park Y. S. (2016). Testing and Assessments of Persons and Communities of Color. Washington, DC: American Psychological Association.

[ref44] Lowden K. Hall S. Elliot D. Lewin J. (2011). Employers’ perceptions of the employability skills of new graduates. Available at: https://www.educationandemployers.org/wp-content/uploads/2014/06/employability_skills_as_pdf_-_final_online_version.pdf (Accessed May 23, 2019).

[ref45] Lunenburg F. C. (2011). Self-efficacy in the workplace: implications for motivation and performance. Int. J. Manag. Bus. Adm. 14, 1–6.

[ref46] Luthans F. Avolio B. J. (2007). Positive psychological capital: measurement and relationship with performance and satisfaction. Pers. Psychol. 60, 541–572. doi: 10.1111/j.1744-6570.2007.00083.x

[ref47] Masitoh L. F. Fitriyani H. (2018). Improving students’ mathematics self-efficacy through problem learning. Malikussaleh J. Math. Learn. 1, 26–30. doi: 10.29103/mjml.v1i1.679

[ref48] Mazaheri S. H. Yazdani S. (2016). A comparison of self-efficacy and oral presentation ability between TEFL students in the class. Int. J. Human. Cult. Stud. 2009–2025.

[ref49] McMahon M. Patton W. Tatham P. (2003). Managing life, learning and work in the twenty-first century. Australian Blueprint for Career Development. 1–17. Available at: http://hdl.voced.edu.au/10707/68613 (Accessed June 20, 2020).

[ref50] Mlambo V. H. Mlambo D. N. Adetiba T. C. (2021). Expansion of higher education in South Africa: problems and possibilities. J. Soc. Soc. Anthropol. 12, 30–40. doi: 10.31901/24566764.2021/12.1-2.363

[ref51] Mlatsheni C. (2012). The challenges of unemployment imposes on youth. Shaping the Future of South Africa’s Youth: Rethinking Post-school Education and Skills Training, 31–41. Cape Town: Centre for Higher Education Transformation/African Minds. Available at: http://www.africanminds.co.za/wp-content/uploads/2012/06/13407164351047234866.pdf (Accessed November 16, 2021).

[ref52] Mohamad N. Dahlan A. Talmizie M. Rizman Z. I. Rabi’ah N. H. (2013). Automated ICT literacy skill assessment using rateskill system. Int. J. Sci. Res. 2, 190–195.

[ref53] Moolman H. (2017). A conceptual competence-based framework for enhancing the employability of graduates. Indep. J. Teach. Learn. 12, 26–43. doi: 10.10520/EJC-c4e375d2f

[ref54] Morrison A. (2014). A class act: lecturers’ views on undergraduates’ employability. Br. J. Sociol. Educ. 35, 487–505. doi: 10.1080/01425692.2013.802420

[ref55] Moynihan L. M. Roehling M. V. LePine M. A. Boswell W. R. (2003). A longitudinal study of the relationships among job search self-efficacy, job interviews, and employment outcomes. J. Bus. Psychol. 18, 207–233. doi: 10.1023/A:1027349115277

[ref56] Mulder G. Taylor N. (2015). Validity of Assessment Centres (ACs) as a Selection or Development Measure. Nari: JvR Psychometrics, South Africa.

[ref57] Muraraneza C. Mtshali N. G. F. Mukamana D. (2017). Issues and challenges of curriculum reform to competency-based curricula in Africa: A meta-synthesis. Nurs. Health Sci. 17, 5–12. doi: 10.1111/nhs.12316, PMID: 27805792

[ref58] National Planning Commission (2012). National development plan 2030 [Executive Summary]. Available at: https://www.gov.za/sites/default/files/Executive%20Summary-NDP%202030%20-%20Our%20future%20-%20make%20it%20work.pdf (Accessed November 16, 2021).

[ref59] Ngo H. Y. Liu H. Cheung F. (2017). Perceived employability of Hong Kong employees: its antecedents, moderator and outcomes. Pers. Rev. 46, 17–35. doi: 10.1108/PR-01-2015-0015

[ref60] Okeke-Uzodike O. E. Naude M. (2018). The perceived work-readiness of supply chain university graduates at a large FMCG company. J. Contemp. Manag. 15, 424–446. doi: 10.10520/EJC-152693ac05

[ref61] Öncel L. (2014). Career adapt-abilities scale: convergent validity of subscale scores. J. Vocat. Behav. 85, 13–17. doi: 10.1016/j.jvb.2014.03.006

[ref62] Oriol-Granado X. Mendoza-Lira M. Covarrubias-Apablaza C. G. Molina-López V. M. (2017). Positive emotions, autonomy support and academic performance of university students: The mediating role of academic engagement and self-efficacy. Revista de Psicodidáctica 22, 45–53. doi: 10.1387/RevPsicodidact.14280

[ref63] Pajares F. (2003). Self-efficacy beliefs, motivation, and achievement in writing. A review of literature. Read. Writ. Q. 19, 139–158. doi: 10.1080/10573560390143085

[ref64] Paton D. Jackson D. (2002). Developing disaster management capability: An assessment Centre approach. Disast. Prevent. Manag. 11, 115–122. doi: 10.1108/09653560210426795

[ref65] Petruzziello G. Mariani M. G. Chiesa R. Guglielmi D. (2021). Self-efficacy and job search success for new graduates. Pers. Rev. 50, 225–243. doi: 10.1108/PR-01-2019-0009

[ref66] Pinquart M. Juang L. P. Silbereisen R. K. (2003). Self-efficacy and successful school- to-work transition: A longitudinal study. J. Vocat. Behav. 63, 329–346. doi: 10.1016/S0001-8791(02)00031-3

[ref67] Republic of South Africa (1974). Health Professions Act, No. 65 of 1974. Pretoria: Government Printers. Available at: https://www.hpcsa.co.za/Uploads/Legal/legislation/health_professions_ct_56_1974.pdf (Accessed November 16, 2021).

[ref68] Republic of South Africa (1998). Employment Equity Act, No. 55 of 1998. Pretoria: Government Printers. Available at: http://www.labour.gov.za/DocumentCenter/Acts/Employment%20Equity/Act%20-%20Employment%20Equity%201998.pdf (Accessed November 16, 2021).

[ref69] Roberts M. (2018). The in-basket exercise and how to use it. The Balance Careers. Available at: https://www.thebalancecareers.com/in-basket-exercise-1669403 (Accessed July 06, 2019).

[ref70] Saadat S. Kord N. Jalali M. (2019). The role of psychological capital (self-efficacy, hope, resilience, and optimism) in student achievement motivation. Rooyesh-e-Ravanshenasi J. Psychol. 8, 167–174.

[ref71] Saeid N. Eslaminejad T. (2017). Relationship between students’ self-directed-learning readiness and academic self-efficacy and achievement motivation in students. Int. Educ. Stud. 10, 225–232. doi: 10.5539/ies.v10n1p225

[ref72] Sanchitra V. Bandara U. (2017). Measuring the academic self-efficacy of undergraduates: The role of gender and academic year experience. Int. Scholar. Sci. Res. Innov. 11, 1–6. doi: 10.5281/zenodo.1132491

[ref73] Schippmann J. S. Prien E. P. Katz J. A. (1990). Reliability and validity of in-basket performance measures. Pers. Psychol. 43, 837–859. doi: 10.1111/j.1744-6570.1990.tb00685.x

[ref74] Scholz U. Gutiérrez Doña B. Sud S. Schwarzer R. (2002). Is general self-efficacy a universal construct?: psychometric findings from 25 countries. Eur. J. Psychol. Assess. 18, 242–251. doi: 10.1027//1015-5759.18.3.242

[ref75] Schwarzer R. (2014). Everything you wanted to know about the general self-efficacy scale but were afraid to ask. 1–8. Berlin, Germany: Freie Universitat. Available at: http://userpage.fu-berlin.de/%7Ehealth/faq_gse.pdf (Accessed June 22, 2019).

[ref76] Schwarzer R. Jerusalem M. (1995). “Generalized self-efficacy scale,” in Measures in Health Psychology: A user’s Portfolio. Causal and Control Beliefs. eds. Weinman J. Wright S. Johnston M. (Windsor, UK: NFER-NELSON), 35–37.

[ref77] Shokhmgar Z. Mohammadpour M. Sanjari M. (2018). Effectiveness of self-efficacy group training on achievement motivation and self-efficacy of high school students. J. Clin. Basic Res. 2, 32–38. doi: 10.29252/jcbr.2.1.32

[ref78] Simons J. C. Buitendach J. H. (2013). Psychological capital, work engagement and organizational commitment amongst call Centre employees in South Africa. SA J. Ind. Psychol. 39, 1–12. doi: 10.4102/sajip.v39i2.1071

[ref79] Smith C. S. Hung L. (2017). Using problem-based learning to increase computer self-efficacy in Taiwanese students. J. Interact. Learn. Environ. 25, 329–342. doi: 10.1080/10494820.2015.1127818

[ref80] Soares I. S. S. Dias D. Monteiro A. Proenca J. F. (2017). “Learning outcomes and employability: A case study on management academic programmes [Paper presentation].” in *International Technology, Education and Development Conference*; March 6-8, 2017; Valencia, Spain.

[ref81] Statistics Solutions (2019). The Wilcoxon Sign Test in SPSS. Available at: https://www.statisticssolutions.com/the-wilcoxon-sign-test-in-spss/ (Accessed March 22, 2019).

[ref82] Statistics South Africa (2021). Quarterly Labour Force Survey—Q2:2021. Available at: http://www.statssa.gov.za/?p=12121 (Accessed November 16, 2021).

[ref83] Stevens B. Hyde J. Knight R. Shires A. Alexander R. (2017). Competency-based training and assessment in Australian postgraduate clinical psychology education. Clin. Psychol. 21, 174–185. doi: 10.1111/cp.12061

[ref84] Strudwick L. (2017). Training for Assessors: A Collection of Activities for Training Assessment Centre Assessors, Role Players and Resource Persons. New York (NY), USA: Routledge, 1–384.

[ref85] Sukalova V. Hraskova D. (2006). Assessment and development centre in human resource management. Vadyba Manag. 1, 97–100. doi: 10.4324/9781315235813

[ref86] Sulla V. Zikhali P. (2018). Overcoming poverty and inequality in South Africa: An assessment of drivers, constraints and opportunities, 1–148. Washington, DC: World Bank.

[ref87] Tay C. Ang S. Van Dyne L. (2006). Personality, biographical characteristics, and job interview success: A longitudinal study of the mediating effects of interviewing self-efficacy and the moderating effects of internal locus of causality. J. Appl. Psychol. 91, 446–454. doi: 10.1037/0021-9010.91.2.446, PMID: 16551195

[ref88] The Skills Portal (2018). Engaging graduate students through career development support. Available at: https://www.skillsportal.co.za/content/engaging-graduate-students-through-career-development-support (Accessed February 26, 2019).

[ref89] Thornton G. C. Rupp D. E. Hoffman B. (2015). Assessment Center Perspectives for Talent Management Strategies. New York: Routledge.

[ref90] Tomlinson M. Nghia T. L. H. (2020). “An overview of the current policy and conceptual landscape of graduate employability,” in Developing and Utilizing Employability Capitals. 1st Edn. eds. Nghia T. L. H. Pham T. Tomlinson M. Medica K. Thompson C. D. (New York (NY): Routledge).

[ref91] Tremblay K. Lalancette D. Roseveare D. (2012). Assessment of higher education learning outcomes: Feasibility study report, volume 1 design and implementation. Organisation for Economic Co-operation and Development. 9–192. Available at: http://hdl.voced.edu.au/10707/241317 (Accessed November 16, 2021).

[ref92] Van Wyk S. du Toit R. (2018). Behaviour-based assessments in the special forces environment: A procedural review. South Afr J. Military Stud. 46, 109–126. doi: 10.5787/46-2-1154

[ref93] Warech M. A. (2002). Competency-based structured interviewing at the Buckhead beef company. Cornell Hotel Restaur. Admin. Q. 43, 70–77. doi: 10.1016/S0010-8804(02)80010-6

[ref94] Wijnia L. Loyens S. M. Derous E. (2011). Investigating effects of problem-based versus lecture-based learning environments on student motivation. Contemp. Educ. Psychol. 36, 101–113. doi: 10.1016/j.cedpsych.2010.11.003

[ref95] Willis J. (2007). Brain-Friendly Strategies for the Inclusion Classroom. Alexandria, VA: ASCD, 1–229.

[ref96] Xing B. Marwala T. (2017). Implications of the fourth industrial age on higher education.The_Thinker_Issue_73__Third_Quarter_2017. Available at: https://arxiv.org/pdf/1703.09643 (Accessed September 12, 2019).

[ref97] Yorke M. (2006). Employability in Higher Education: What it Is—What it Is Not. Learning and Employability Series. York: Higher Education Academy.

[ref98] Zimmerman B. (1990). Self-regulating academic learning and achievement. Educ. Psychol. Rev. 2, 173–201. doi: 10.1007/BF01322178

